# Efficacy of Pulmonary Rehabilitation in Post-COVID-19: A Systematic Review and Meta-Analysis

**DOI:** 10.3390/biomedicines11082213

**Published:** 2023-08-07

**Authors:** Erika Meléndez-Oliva, Oliver Martínez-Pozas, Juan Nicolás Cuenca-Zaldívar, Jorge Hugo Villafañe, Laura Jiménez-Ortega, Eleuterio A. Sánchez-Romero

**Affiliations:** 1 Department of Physiotherapy, Faculty of Sport Sciences, European University of Valencia, Pg. de l’Albereda, 7, 46010 Valencia, Spain; erikamelendezoliva@gmail.com; 2 Interdisciplinary Group on Musculoskeletal Disorders, Faculty of Sport Sciences, Universidad Europea de Madrid, 28670 Villaviciosa de Odón, Spain; 3 Physiotherapy and Orofacial Pain Working Group, Sociedad Española de Disfunción Craneomandibular y Dolor Orofacial (SEDCYDO), 28009 Madrid, Spain; 4 Department of Physical Therapy, Occupational Therapy, Rehabilitation and Physical Medicine, Escuela Internacional de Doctorado, Universidad Rey Juan Carlos, 28933 Alcorcón, Spain; 5 Grupo de Investigación en Fisioterapia y Dolor, Departamento de Enfermería y Fisioterapia, Facultad de Medicina y Ciencias de la Salud, Universidad de Alcalá, 28801 Alcalá de Henares, Spain; 6 Research Group in Nursing and Health Care, Puerta de Hierro Health Research Institute-Segovia de Arana (IDIPHISA), 28222 Majadahonda, Spain; 7 Physical Therapy Unit, Primary Health Care Center “El Abajón”, 28231 Madrid, Spain; 8 IRCCS Fondazione Don Carlo Gnocchi, 20148 Milan, Italy; mail@villafane.it; 9 Department of Psychobiology and Behavioral Sciences Methods, Faculty of Odontology, Complutense University of Madrid, 28040 Madrid, Spain; laurajim@ucm.es; 10 Center of Human Evolution and Behavior, Carlos III Health Institute, Complutense University of Madrid, 28040 Madrid, Spain; 11 Department of Physiotherapy, Faculty of Sport Sciences, European University of Madrid, Villaviciosa de Odón, 28670 Madrid, Spain

**Keywords:** COVID-19, pulmonary rehabilitation, exercise

## Abstract

Background: This systematic review and meta-analysis examines how pulmonary rehabilitation impacts in patients suffering from subacute and long COVID-19 infections, gauging enhancements in of dyspnea, physical function, quality of life, psychological state (anxiety and depression), and fatigue. Methods: Three electronic databases (PubMed, Web of Science, Cochrane Library) were systematically searched for full-text articles published from inception to January 2023. Randomized, quasi-experimental, and observational studies were included, with adults diagnosed with subacute or long COVID-19 who received pulmonary rehabilitation as intervention. Outcomes related to dyspnea, physical function, quality of life, fatigue, and psychological status were included. Risk of bias was assessed with Cochrane Risk of Bias Tool for Randomized Controlled Trials and Risk of bias in non-randomized studies of intervention. The review was registered before starting in PROSPERO (CRD: 42022373075). Results: Thirty-four studies were included, involving 1970 patients with subacute and long COVID-19. The meta-analysis demonstrated moderate to large effects on dyspnea, physical function, quality of life, and depressive symptoms compared to usual care intervention. No significant differences were found in fatigue compared to usual care, nor in anxiety levels after pulmonary rehabilitation intervention. Conclusions: Pulmonary rehabilitation has the potential to improve health outcomes in patients with subacute and long COVID-19. However, due to the high risk of bias of included studies, conclusions should be taken with caution.

## 1. Introduction

COVID-19 is a disease caused by infection with the highly contagious SARS-CoV-2 virus, whose main condition falls on the respiratory system, with symptoms such as dyspnea, fibrosis, and pulmonary edema, but which also presents a wide variety of comorbidities such as musculoskeletal pain, fatigue and muscle weakness, cardiovascular and cerebrovascular diseases, and psychological conditions such as depression and anxiety, deteriorating seriously, in most cases, the quality of life [1,2]. 

Currently, it can be argued that the severity of COVID-19 has decreased in many countries, possibly due to the wide vaccination coverage carried out worldwide and effective treatment [3]. However, many patients affected by COVID-19 continue to experience symptoms after the acute phase, such as breathlessness, fatigue, neuropsychological symptoms, cough, and musculoskeletal pain [4,5]. This post-acute syndrome is known as long COVID (LC), and it is defined by WHO as “the continuation or development of new symptoms three months after the initial SARS-CoV-2 infection” [6]. 

Pulmonary rehabilitation (PR) is a very effective exercise-based therapeutic strategy to improve functional capacity, dyspnea, and health-related quality of life in patients with chronic obstructive disease [7]. 

PR has been shown to improve the physical and psychological well-being of patients with COVID-19 [8]. This is achieved through aerobic endurance and resistance training, which help increase muscle mass and strength, especially in peripheral muscles [9,10]. In addition, PR can incorporate thoracic mobility exercises to improve lung expansion.

Previous studies [11,12] have reported that supervised PR programs are safe and effective in improving exercise capacity, lung function, exertional dyspnea, psychological well-being, and quality of life in patients with COVID-19. In addition, PR has been shown to significantly reduce the frequency and duration of hospital stays in individuals with restrictive lung disease [13].

Furthermore, several systematic reviews have recently been published showing that also leads to enhancements in both physical and pulmonary capabilities among patients with acute and subacute COVID-19 [14,15,16,17,18]. 

The main objective of this systematic review with meta-analysis is to provide an update about the efficacy of PR in patients with subacute and long COVID-19 (LC), and its effects on dyspnea, physical function, quality of life, psychological outcomes, and fatigue. 

## 2. Materials and Methods

This systematic review was conducted by the Preferred Reporting Items for Systematic Reviews and Meta-Analysis (PRISMA) [19] and was registered before starting in PROSPERO (CRD42022373075).

### 2.1. Search Strategy

The literature search strategy involved structured searches of PubMed, Web of Science and Cochrane Library for relevant articles published from inception to January 2023. Reference lists of studies were reviewed for potential additional references not identified in the primary search, and the authors were contacted for further information if necessary. No language filters were applied to retrieve all potentially eligible studies, as recommended by international criteria [20]. 

The search terms combined medical subject headings (MeSH terms) and non-MeSH terms, adding a Boolean operator (OR and/or AND) to combine them. MeSH terms used were some such as “Rehabilitation”, “Exercise”, or “COVID-19” among other non-MeSH term such as “Pulmonary Rehabilitation”, “Long-Covid”, or “Post-acute COVID-19”. The complete search strategy can be found in Appendix A, which shows the PubMed search strategy, which was adjusted for other databases if necessary. 

### 2.2. Eligibility Criteria

The study population includes studies with adults (18 years or older) diagnosed with sub-acute or long COVID-19 (LC), being patients with symptoms persisting for less than three months considered sub-acute and those with symptoms lasting more than three months considered LC [6]. Studies with acute patients, with positive testing, were excluded. 

The intervention consisted of PR, defined as “interventions based on, but not limited to, exercise training, education and behavior change designed to improve physical and psychological conditions of people with respiratory diseases” [21]. Both strategies, face-to-face and telerehabilitation, were included as PR. 

Outcomes related to dyspnea, physical function, quality of life, fatigue, and psychological status were included. 

Randomized controlled trials (RCTs), non-randomized controlled trials (quasi-experimental studies), and observational studies were eligible for inclusion in the review if they met the inclusion criteria.

### 2.3. Inclusion Procedure

Titles and abstracts were screened manually and independently by two authors (Oliver Martínez-Pozas, Erika Meléndez-Oliva) and any disagreements between reviewers were resolved by consensus or, if needed, by a third member (Eleuterio A. Sánchez-Romero). Articles were excluded if they: included patients with positive COVID-19 (acute phase) or hospitalized patients, or included patients who did not receive PR. To minimize the risk of investigator bias, all investigators had to agree on whether each study met all the eligibility criteria. The inclusion procedure included a first phase based on the study’s title, abstract, and keywords. Subsequently, the studies were evaluated in their entirety to assess their potential eligibility according to inclusion criteria. The data were extracted by two researchers (Oliver Martínez-Pozas and Erika Meléndez-Oliva) and the data described in the Results section were extracted using a structured protocol that ensured that the most relevant information was obtained from each [22]. 

### 2.4. Risk of Bias Assessment

Two independent reviewers assessed the risk of bias in included studies (Oliver Martínez-Pozas, Erika Meléndez-Oliva) and disagreements were resolved through consensus and/or mediation by a third reviewer (Eleuterio A. Sánchez-Romero). The risk of bias of RCTs was evaluated using the “Revised Cochrane Risk of Bias Tool for Randomized Controlled Trials (RoB 2.0)” which contains five domains: bias arising from the randomization process, bias due to deviation from intended interventions, bias due to missing outcome data, bias in the measurement of the outcome, and bias in the selection of the reported result [23]. Each domain was scored as “low risk”, “some concerns”, and “high risk”, and each study was classified into one of three categories as “high risk of bias”, “some concerns”, or “low risk of bias” [23]. 

Risk of bias in non-randomized controlled trials was evaluated using the “Risk of bias in non-Randomized studies of intervention” (ROBINS-I) which evaluates domains such as confounding factors, selection of participants, classification of interventions, deviations from the intended interventions, missing data, measurements of outcomes, and selection of the reported results [24]. Each study was classified into five categories as “low risk of bias”, “moderate risk of bias”, “serious risk of bias”, “critical risk of bias”, or “no information” [24].

### 2.5. Data Analysis

For the statistical analysis, the program R version 4.1.3 (R Foundation for Statistical Computing, Institute for Statistics and Mathematics, Welthandelsplatz 1, 1020 Vienna, Austria) was used. Meta-analysis was conducted using the metafor and meta r packages [25,26].

In the articles in which the results were shown with median and with maximum and minimum or interquartile range, these were transformed into mean and standard deviation using the appropriate formulas [27,28]. 

In the RCTs, a meta-analysis of pre-post-intervention change was performed, analyzing the level of significance between the treatment and control groups using the standardized mean difference (SMD). Since no study reported the pre–post intervention mean ± standard deviation of change, these were calculated using the following formulas [29]:

(1) Meanchange=Meanfinal−Meanbaseline

(2) $$SDchange=\sqrt{{SD}^{2}baseline+{SD}^{2}final-(2\cdot r\cdot SDbaseline\cdot SDfinal)}\;$$

In the formulas, *SD* is the standard deviation and “*r*” is the pre–post intervention correlation coefficient which, since the standard deviation of the change was not reported, was assigned a value of 0.7 in order to obtain a conservative estimate, [30] as has been done in other works [31,32,33,34,35]. 

In the study by Jimeno-Almanzán 2022 [36] in which, for the quality-of-life outcome, three interventions are reported against the control, these were sequentially combined using the appropriate formulas [29]. 

In observational studies, a single group meta-analysis was performed with the mean change pre-post intervention in each study. 

In both cases a random effects model was applied given the heterogeneity between the studies. Heterogeneity was tested by estimating the between-study variance τ^2^ (calculated with the DerSimonian–Laird estimator with Hartung–Knapp correction) using the Cochran Q test as well as the I^2^ estimator. The latter being defined as not important (<30%), moderate (30–50%), large (50–75%), and important (>75%) heterogeneity.

Effect sizes were calculated in RCTs with Hedge’s g being defined as small (<0.2), moderate (0.2–0.8), and large (>0.8).

Heterogeneity was assessed by detecting those outlier studies with absolute values in the standardized residuals greater than 3. In addition, a sensitivity analysis was performed using the leave-one-out method. The contribution of individual studies to heterogeneity was assessed using a Baujat plot showing the contribution of each study to heterogeneity calculated with Cochran’s Q test versus its influence on the overall effect of the meta-analysis [37]. Subgroup meta-analyses were also performed to explore the heterogeneity detected based on the type of test used in each of the outcome variables.

Finally, publication bias was analyzed using trim and fill funnel plots and a Begg and Egger’s test [38].

## 3. Results

**Figure 1:**
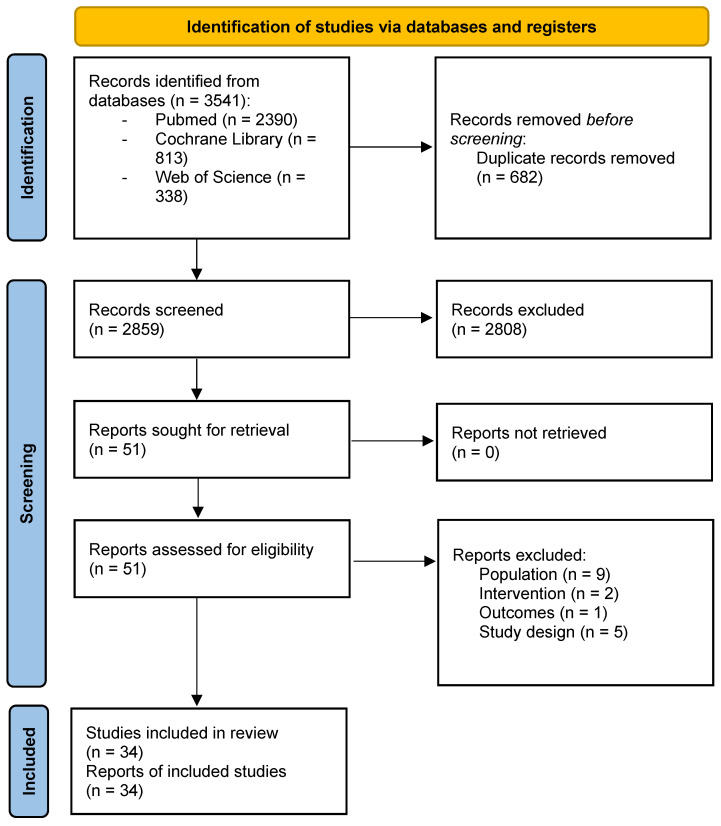
PRISMA 2020 Flowchart.

Database searching reported 3541 articles among different databases. After screening for title and abstract and removing duplicates, 51 studies were assessed for eligibility. Nine studies were excluded due to population (included patients with acute COVID-19, still testing positive in COVID-19 test), two were excluded due to intervention (robot devices, ventilation therapy), one study was excluded due to outcomes (salivary biomarkers), and five due to study design (congress abstract, protocol). Finally, 34 studies were included for qualitative analysis in the present review (20 RCTs and 14 observational studies) [36,39,40,41,42,43,44,45,46,47,48,49,50,51,52,53,54,55,56,57,58,59,60,61,62,63,64,65,66,67,68,69,70,71]. For quantitative analysis, 26 studies were included. The flowchart of included studies can be found in Figure 1. In addition, PRISMA Checklist can be found in Supplementary Materials (Table S1).

### 3.1. Characteristics of the Included Studies

The present systematic review included 34 studies, with a total of 1970 adults. All participants were adults diagnosed with sub-acute COVID-19 (*n* = 18 studies) or LC (*n* = 16 studies). The number of patients for studies varied from 23 to 150. The average age of patients varied from 32 to 82 years. The characteristics of the included, including data from overall population analyzed, are shown in Table 1.

### 3.2. Intervention

Most of the included studies carried out performed PR programs based on exercise and breathing retraining as the main components of PR, varying in the number of sessions and intervention approaches employed.

Isolated breathing exercises were used in seven studies, five performed via telerehabilitation [42,58,62,68,71], and two face-to-face [39,40]. Breathing exercises were performed in addition to myofascial release in two studies [41,50]. Breathing exercises were performed with handheld devices, breathing control exercises or secretion mobilization exercises.

Exercise therapy in addition to breathing exercises was performed in ten studies [36,44,45,46,52,54,59,63,69,70]. Isolation exercise was used in ten studies [47,49,51,55,56,61,64,65,66,67]. One study used exercise therapy in addition to psychological therapy [60]. The different studies included a combination of aerobic and strength exercises, while two of them incorporated virtual reality as part of the exercise regimen [55,64].

In four studies, a multicomponent program with exercise, breathing training, psychological counseling, and nutritional advice was performed [43,48,53,57].

Regarding how PR was administered, 15 studies included telerehabilitation protocols, while the remaining were administered face-to-face.

### 3.3. Outcomes

The main outcomes measured were dyspnea, physical function, quality of life, psychological outcomes, and fatigue. However, high heterogeneity was found when measuring outcomes, with different scales measuring the same outcome.

The Modified Medical Council Research Scale was the most commonly used test for the assessment of dyspnea, followed by Borg Scale and Modified Borg Scale. Other scales used were the Dyspnea-12 questionnaire, Multidimensional Dyspnea 12, Visual Analogue Scale or Transition Dyspnea Index.

Regarding physical function, the 6-min walking test was the most used test to assess functional capacity. Other tests used were the 30-s sit-to-stand test, time up and go test, short physical performance battery, or 1-min sit-to-stand test.

Quality of life was analyzed with different scales, but Euroqol 5D was the most widely used, followed by SF-12 and SF-36. Other studies used the Saint George Respiratory Questionnaire, SarQol, WHOQOL-BREEF, and K-BILD scales.

Anxiety was usually analyzed with the Hospital Anxiety and Depression Scale and Generalized Anxiety Disorder-7 scale, and to a lesser extent with the Self-Rating Anxiety Scale and C19YRS Scale. Depressive symptoms were commonly evaluated with Hospital Anxiety and Depression Scale and with Patient Health Questionnaire-9, and to a lesser extent with the Self-Rating Depression Scale, Beck Depression Inventory, and C19YRS.

Finally, fatigue was the most heterogeneous outcome, as all studies reported different scales, such as Fatigue Assessment Scale, Borg Scale, Fatigue Severity Scale, or Brief Fatigue Inventory.

In addition, inverse scales were assessed among the included studies, which was considered when performing the meta-analysis. For example, when assessing physical function, higher scores on the 6MWT were related to higher physical function, while lower scores on 5-time sit-to-stand test were related to higher physical function.

### 3.4. Risk of Bias

**Figure 2:**
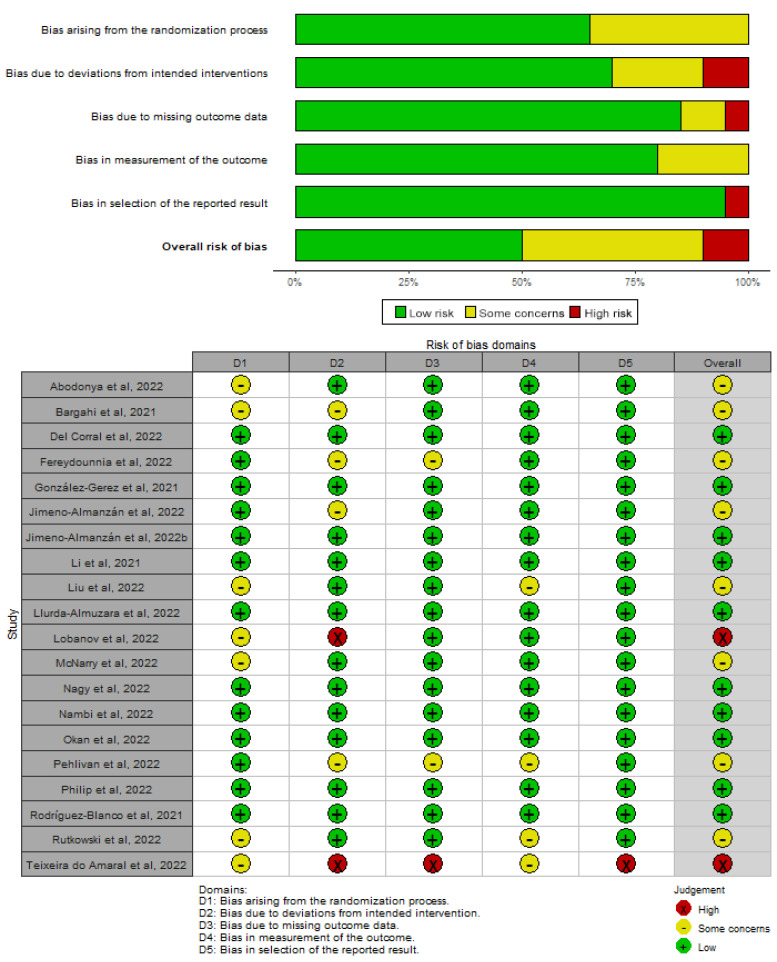
Risk of bias of randomized controlled trials (RoB 2.0) [36,39,40,41,42,45,46,47,48,50,51,52,54,55,62,66,68,70,71].

The risk of bias of RCTs ranged from low to high, with ten studies with low risk of bias [42,45,47,50,51,54,62,66,70,71], eight with some concerns [36,39,40,41,46,52,55,68], and two with high risk [48,56]. Domains of bias due to the randomization process and bias due to deviation from the intended interventions were the domains with higher issues, while the domain related to the selection of the reported results was the domain with better scores. The quality of evidence of RCTs can be found in Figure 2.

**Figure 3:**
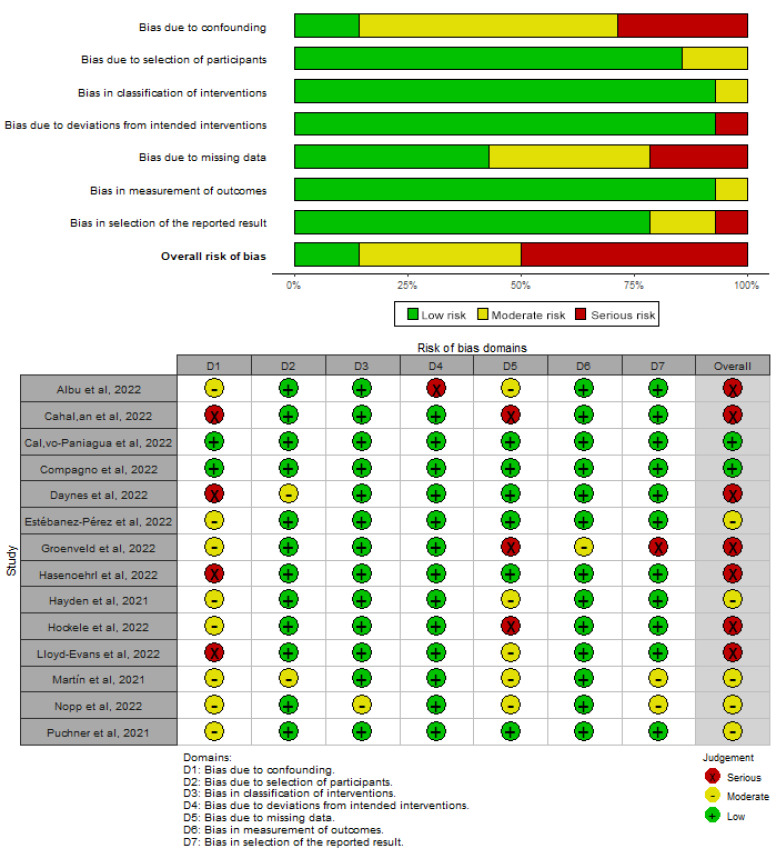
Risk of bias of nonrandomized controlled trials of intervention (ROBINS-I) [43,44,49,53,57,58,59,60,61,63,64,65,67,69].

Regarding observational studies, risk of bias ranged from low to serious risk of bias. Only two studies had a low risk of bias [59,60], while five studies had a moderate risk of bias [43,49,53,63,69] and seven had serious risk [44,57,58,61,64,65,67]. The domain of controlling for confounding factors was the most critical domain of all included studies, with only two studies controlling the main factors (such as ICU stay/length, and pre-existing comorbidities). Domain of missing data was another critical domain, with many studies reporting outcomes biased due to the high loss of patients. Quality of evidence of observational studies can be found in Figure 3.

### 3.5. Efficacy of Pulmonary Rehabilitation in COVID-19

**Figure 4:**
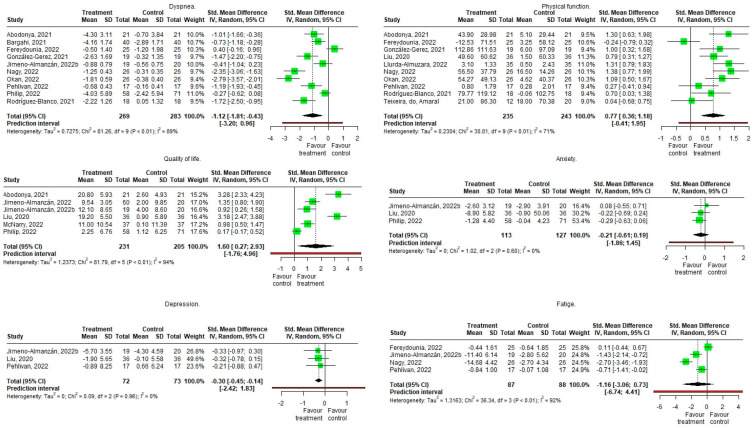
Meta-analysis (forest plot) of Randomized Controlled Trials on the effect of Pulmonary Rehabilitation in patients suffering from subacute and long COVID-19 infections, gauging enhancements in of dyspnea, physical function, quality of life, psychological state (anxiety and depression), and fatigue [36,39,40,41,42,45,46,47,48,50,51,52,54,55,56,62,66,68,70,71].

In the RCTs a significant effect is observed with a higher pre-post intervention change in the treatment group in dyspnea with a large effect size (Hedge’s g = −1.12 [−1.813, −0.427], Z =−3.656, *p* = 0.005), in physical function with a moderate effect size (Hedge’s g = 0.771 [0.363, 1.178], Z = 4.276, *p* = 0.002), on quality of life with a large effect size (Hedge’s g = 1.6 [0.266, 2.934], Z = 3.083, *p* = 0.027), and at the level of depression with a moderate effect size (Hedge’s g = −0.295 [−0.446, −0.145], Z = −8.432, *p* = 0.014) without significant effects on the level of anxiety or fatigue perceived by the patients, although the effects in both occur in favor of the group of patients. The heterogeneity of the studies is important in dyspnea (I^2^ = 89%), fatigue (I^2^ = 92%), and quality of life (I^2^ = 94%); moderate physical function (I^2^ = 71%); and null in depression and anxiety (I^2^ = 0%) (Figure 4).

**Figure 5:**
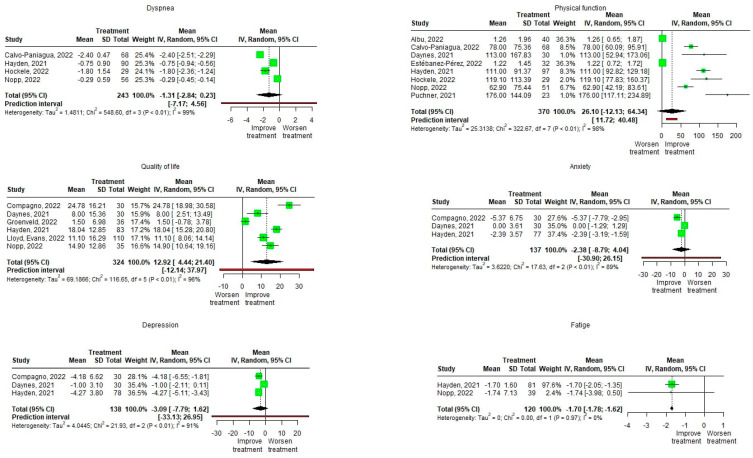
Meta-analysis (forest plot) of Observational Studies on the association of Pulmonary Rehabilitation in patients suffering from subacute and long COVID-19 infections, and improvements in dyspnea, physical function, quality of life, psychological state (anxiety and depression), and fatigue [43,44,49,53,57,58,59,60,61,63,64,65,67,69].

In the case of observational studies, significant effects are observed in the pre-post-intervention change in quality of life with an improvement in quality of life (Mean = 12.916 [4.438, 21.395]) and on the level of perceived fatigue, with a decrease in it (Mean = −1.701 [−1.778, −1.624]), without producing significant changes in dyspnea, physical function, or in the level of anxiety and depression, although their effects show an improvement in the patients. The heterogeneity of the studies is important in dyspnea (I^2^ = 99%), physical function (I^2^ = 98%), quality of life (I^2^ = 96%), anxiety (I^2^ = 89%), and depression (I^2^ = 91%), and null in the level of perceived fatigue (I^2^ = 0%) (Figure 5).

### 3.6. Subgroup Analysis

The subgroups meta-analysis in the RCTs (Supplementary Materials, Figure S1) showed that for dyspnea only, the studies using the Borg Scale (BS) scale show a significant and large decrease in dyspnea levels for the treatment group (Hedge’s g = −1.59 [−3.161, −0.02], Z = −12.865, *p* =0.049) and with vanishing heterogeneity (I^2^ reduced from 89% to 0%).

In physical function, studies using the 6 MWT are the one showing a significant and moderate effect in favor of the treatment group (Hedge’s g = 0.756 [0.269, 1.242], Z = 3.673, *p* = 0.008) with an unchanging heterogeneity (I^2^ of the 71% that persists in the 71%).

Regarding quality of life, only the study by Abodonya et al. [39] found a significant large reduction in the EQ−5D scale scores (Hedge’s g = 3.276 [2.325, 4.228], Z = 6.751, *p* = 0), while, even without having a significant effect, studies using the SF-12 Physical scale were the ones presenting the smallest heterogeneity (I^2^ from 94% which is reduced to 0%).

Finally, in the case of the level of depression, the three studies use different scales, so the impact on heterogeneity could not be evaluated based on the evaluation type.

The subgroups meta-analyses in the observational studies (Supplementary Materials, Figure S2), regarding the quality of life, a study by Compagno et al. [60] using SF-36 Physical scale shows a pre-post treatment significant increase in the scores for the patient’s group (Mean = 24.78 [18.979, 30.581]). Similarly, in studies using the EQ-5D, significant increases in quality of life were observed (Mean = 13.304 [6.417, 20.19]), as well as a reduction in heterogeneity (I^2^ from 96% to 82%).

Regarding fatigue, the study by Hayden et al. [43] uses the BFI scale observing pre-post treatment significant score decreases (Mean = −1.7 [−2.048, −1.352]).

In the variables without significant effects, however, a notable enhancement in physical function was observed in patients evaluated using the 6MWT (Mean = 101.188 [52.588, 149.788]) with a reduction in heterogeneity (I^2^ of the 98% which reduces to the 83%); the study by Compagno et al. [60] assessed anxiety and depression levels with the SAS scale where significant reductions were observed for both scales (Mean = −5.37 [−7.785, −2.955]; Mean = −4.18 [−6.55, −1.81] for anxiety and depression, respectively).

### 3.7. Heterogeneity Analysis

Both in the RCT and in the observational studies with significant effects, no outlier study was detected (Supplementary Materials, Figures S3 and S4).

The leave-one-out analysis in the RCTs shows a stable line in dyspnea, indicating that all studies equally influenced the meta-analyses. Meanwhile, in the case of physical function, the Fereydounia et al. [41] study is the one that exerts the most influence on the results. Regarding quality of life, it is the studies by Abodonya et al. [39], Liu et al. [46], and Philip et al. [71] that most influence the results. Finally, in the level of depression, Pehlivan et al. [52] is the most influential, decreasing the level of total significance (Supplementary Materials, Figure S5). The analysis of the observational studies shows that Groenveld et al. [64] study is the most influential one in terms of quality of life, while neither of the two considered studies seems to influence the results of the meta-analysis regarding the level of fatigue (Supplementary Materials, Figure S6).

The Baujat plot in the RCTs shows how Fereydounia et al. [41] study on physical function, Philip et al. [71] study on quality of life, and Pehlivan et al. [52] study on the depression are the ones that contribute the most to the heterogeneity detected, while no article was found for dyspnea (Supplementary Materials, Figure S7). In the observational studies, the graphs show that for quality of life, Groenveld et al. [64] study is the principal contributor to the detected heterogeneity, while for fatigue levels there is no evidence of any article (Supplementary Materials, Figure S8).

### 3.8. Publication Bias

The Begg and Egger’s tests are significant in the RCTs of dyspnea and in the case of the Egger test, also in the quality of life, while in the observational studies with significant effects, there is no evidence of publication bias (Supplementary Materials, Table S2). The funnel plots show an asymmetric distribution of the RCTs with physical function and dyspnea (in the latter with a large number of studies outside the limits of significance), which corroborates the presence of publication bias in them (Supplementary Materials, Figure S9). In observational studies with quality of life, the funnel plot shows a symmetric distribution, which corroborates the absence of publication bias (Supplementary Materials, Figure S10).

## 4. Discussion

Based on the review, it has been found that PR has a positive impact on dyspnea, physical function, quality of life, and depressive symptoms when compared to usual care interventions. These improvements were of moderate to large magnitude. Furthermore, PR has been effective in reducing fatigue levels, although no significant differences were observed compared to usual care interventions. However, the review did not uncover any significant changes in anxiety levels resulting from PR.

The results of this systematic review are in line with other previously published systematic reviews [14,15,16,17,18,72,73]. However, while this review identified significant improvements in quality of life and depressive symptoms, others did not observe such effects [14,16,18]. Most systematic reviews included studies that assessed anxiety and depression using the Hospital Anxiety and Depression scale (HADS). While analyzing the questionary, Cosco et al. [74] discovered challenges in distinguishing between anxiety and depression. Consequently, the scale could still serve as a valuable total score indicator of emotional distress. On the other hand, Coyne and van Sonderen argue that additional research is unnecessary, advocating for the abandonment of the scale altogether [75]. It is worth noticing that in the present review, none of the included studies used the HADS for assessing depression. Additionally, recent studies have indicated a deterioration in quality of life and increases depressive symptoms over time in patients following COVID-19 infection [76,77]. In contrast to the aforementioned studies, this review encompassed patients with long COVID-19, including those with elevated levels of depression and poorer quality of life. This inclusion of patients with more severe symptoms may help explain the observed improvements in quality of life and depressive symptoms reported in our review. It is important to consider the unique characteristics and challenges faced by individuals with long COVID-19, as their experiences and outcomes may differ from those with subacute COVID-19. These distinctions could contribute to variations in the findings across different studies. Furthermore, future systematic reviews should deeply consider whether to include studies using HADS or include them contemplating HADS as a total score assessing simply emotional distress.

The findings of this study indicate that PR can contribute to improving the health status of patients following COVID-19 infection. However, it is important to acknowledge that the studies included in the review primarily focused on short-term outcomes and did not have long-term follow-up. Only one study examined long-term outcomes, revealing significant improvements in physical function after six months of follow-up in patients with subacute COVID-19 who underwent PR [45]. Thus, future research should aim to investigate the effects of PR on various long-term outcomes to gain a comprehensive understanding of its benefits in post-COVID-19 patients.

Despite the absence of a standardized protocol for training patients with COVID-19, exercise was consistently incorporated in all the studies analyzed, emphasizing its significance in managing post-COVID-19 conditions. This consistent inclusion of exercise highlights its role as a fundamental component in the overall treatment approach for patients recovering from COVID-19.

Exercise has demonstrated positive effects on the immune system, strength, fatigue, physical conditioning, and muscle dysfunctions associated with lung diseases, ultimately improving symptoms such as dyspnea [4]. Additionally, physical activity has been also associated with reduced levels of depression, anxiety, and mental well-being, regardless of age [78,79,80]. Furthermore, a recent review found that performing physical activity during COVID-19 is associated with less depression and anxiety [81]. Therefore, exercise might have also helped to improve patients’ mental well-being, resulting in the observed reduction in depression levels. However, considering that mental health disorders in patients with COVID-19 showed prevalence rates of 16% in terms of depression or 15% in terms of anxiety [82], and seeing that psychological therapies such as cognitive behavioral therapy had shown positive effects improving anxiety and depression in patients with COVID-19 compared to usual care [83], only a few studies included psychological therapy among their protocols [43,53,57,58,60]. Thus, future studies should incorporate broader multidisciplinary protocols that address both physical and mental health components.

PR plays a crucial role in the rehabilitation of patients who have experienced prolonged hospitalization in the intensive care unit and have undergone mechanical ventilation [84]. It offers significant potential in improving various aspects of post-COVID-19 syndrome, including dyspnea, fatigue, respiratory function, anxiety, depression, and overall quality of life. PR programs have shown promising results in enhancing these outcomes for patients with post-COVID-19 syndrome. However, despite these promising indications, the specific impact of PR programs on respiratory symptoms in patients with post-COVID-19 syndrome remains relatively limited and requires further exploration in the existing literature. More research is needed to investigate the optimal components and duration of PR interventions, as well as their long-term effects on respiratory symptoms and overall pulmonary function in this particular population. These studies will contribute to a deeper understanding of the role of PR in effectively addressing respiratory symptoms in patients recovering from COVID-19.

### 4.1. Strengths and Limitations

To our knowledge, this is the most comprehensive systematic review with meta-analysis and represents the most comprehensive evaluation of the efficacy of PR in patients with COVID-19, encompassing both subacute and LC patients. However, it is important to acknowledge certain limitations that arise from the following aspects. Firstly, only half of the RCTs included in this review were deemed to have a low risk of bias, and merely two out of the fourteen observational studies were classified as having a low risk of bias. Consequently, the results of this review may be prone to bias due to the high risk of bias exhibited in the included studies. Secondly, clinical heterogeneity was observed among the studies, characterized by variations in intervention protocols, duration, and intensity. This heterogeneity can complicate the synthesis and interpretation of the findings, thereby limiting the ability to draw definitive conclusions about the efficacy of PR in patients with COVID-19. Thirdly, heterogeneity was also evident in the assessment of outcomes, with different scales employed to measure the same outcome. For instance, fatigue was evaluated using eight distinct scales across the included studies, which introduces challenges in reaching robust conclusions regarding the effectiveness of PR in improving fatigue. Fourthly, the sample size of the included studies was generally low, and long-term effects of PR were only reported in a single study. Consequently, the limited sample sizes restrict the statistical power and generalizability of the findings, while the absence of long-term data impedes a comprehensive understanding of the sustained benefits of PR in this population.

### 4.2. Clinical Messages

Patients may develop persistent symptoms such as respiratory or physical function impairments, or psychological problems after COVID-19 infection.

Pulmonary rehabilitation has been shown to be effective improving symptoms after COVID-19, including dyspnea, physical function, quality of life, and depressive symptoms compared to usual care.

Future studies with improved methodology and long-term follow-up are needed to strengthen our conclusions.

## 5. Conclusions

In conclusion, the reviewed studies suggest that PR has the potential to improve various health outcomes in patients, including those recovering from COVID-19. PR has shown positive effects on dyspnea, physical function, quality of life, and depressive symptoms when compared to usual interventions. However, it is important to consider the limitations of the existing studies, such as methodological quality and small sizes, which call for mere comprehensive and well-designed research using valid assessment tools. Further investigation is needed to establish stronger evidence regarding the effectiveness of PR and its applicability to patients, particularly those with COVID-19.

## Figures and Tables

**Table 1:** Characteristics of included studies.

Author (Year)	Study Design	Population	Sample Size	Intervention	Control Group	Outcomes	Results
**Studies with Subacute COVID-19 Patients**							
Abodonya et al. (2021) [39]	RCT	Adults with subacute COVID-19	*n* = 42 Int: *n* = 21 (19% F), Age: 48.3 ± 8.5 Con: *n* = 21 (23.8% F) Age: 47.8 ± 9.2	**Duration** 2 weeks. **Intervention** Breathing exercises.	Usual care	Dyspnea (DS-12) Quality of life (EQ-5D) Physical function (6MWT)	Intra-group analysis found statistically differences in intervention group in dyspnea (*p* = 0.001), quality of life (*p* < 0.001) and 6MWT (*p* < 0.001). Between group comparison, intervention group reported statistically significant differences compared to control in all outcomes with medium-large size effects.
Barhagi et al. (2021) [40]	RCT	Adults with subacute COVID-19	*n* = 80 (38.75% F) Int: *n* = 40, Age: 57.1 ± 18.7 Con: *n* = 40 Age: 58 ± 17.13	**Duration** Three days. **Intervention** Breathing exercises.	Usual care	Dyspnea (MBS)	After end of treatment, intervention group improved dyspnea with statistically differences compared to usual care (*p* = 0.007).
Fereydounnia et al. (2022) [41]	RCT	Adults with subacute COVID-19	*n* = 50 (42% F) Int: *n* = 25, Age: 49.44 ± 14.78 Con: *n* = 25, Age: 45 ± 12.75	**Duration** 1 week. **Intervention** Myofascial release and breathing exercises.	Breathing exercises	Dyspnea (MBS) Physical function (6MWT) Fatigue (Borg)	Intervention group improved dyspnea with statistically differences at the end of the treatment compared to control (*p* < 0.01). No statistically differences were found in terms of physical function (*p* = 0.033) or fatigue (*p* = 0.034) improvement compared to control.
González-Gerez et al. (2021) [42]	RCT	Adults with subacute COVID-19	*n* = 38 Int: *n* = 19 (47.4% F), Age: 40.79 ± 9.84 Con: *n* = 19 (42.1% F), Age: 40.32 ± 12.53	**Duration** 1 week. **Intervention** Breathing exercises. Telerehabilitation.	Usual care	Physical function (6MWT; 30STS) Dyspnea (MD12; BS)	Statistically differences were found in terms of improving dyspnea (*p* < 0.001) and physical function (*p* = 0.001), in intervention, with no differences in control group. Between group analysis found statistically differences favoring intervention compared to control improving dyspnea (*p* < 0.001) and physical function (*p* = 0.001).
Hayden et al. (2021) [43]	Observational	Adults with subacute COVID-19	*n* = 108 (45.4% F) Age: 55.6 ± 10.1	**Duration** 3 weeks. **Intervention** Aerobic and strength training. Nutritional, psychological, and physical therapy support were included.	No control	Dyspnea (NRS/mMRC) Physical function (6MWT) Quality of life (EQ-5D) Fatigue (BFI) Depression and Anxiety (PHQ-9, GAD-7)	Dyspnea improved at rest (*p* < 0.001) and on exertion (*p* < 0.001) after treatment. Physical function improved after treatment (*p* < 0.001). Quality of life, fatigue, anxiety, and depression improved after treatment (*p* < 0.001)
Hockele et al. (2022) [44]	Observational	Adults with subacute COVID-19	*n* = 29 (51.7% F) Age: 54.4 ± 14.6	**Duration** 6–8 weeks. **Intervention** Aerobic and strength training.	No control	Physical function (6MWT, TUG) Dyspnea (mMRC)	Physical function improved after treatment with statistically significant differences compared to baseline in 6MWT (*p* < 0.001) and TUG (*p* = 0.023). Dyspnea improved after treatment with differences compared to baseline (*p* = 0.003).
Li et al. (2021) [45]	RCT	Adults with subacute COVID-19	*n* = 119 (55.46% F) Int: *n* = 59, Age: 49.17 ± 10.75 Con: *n* = 60, Age: 52.03 ± 11.10	**Duration** 6 weeks. 6 months follow-up. **Intervention** Aerobic, strength and breathing exercises. Telerehabilitation.	Usual care	Physical function (6 MWT) Dyspnea (mMRC) Quality of life (SF-12)	Intervention group improved physical function after treatment (*p* < 0.001) and at follow-up with statistically differences (*p* < 0.001). Perceived dyspnea improved after treatment with differences compared to control (*p* = 0.001) but without differences at follow-up (*p* = 0.162). Physical component of SF-12 improved with differences after treatment (*p* = 0.004) and at follow-up (*p* = 0.045). However, mental component found no differences at any point (*p* = 0.116; *p* = 0.164).
Liu et al. (2020) [46]	RCT	Adults with subacute COVID-19	*n* = 72 Int: *n* = 36 (33.3% F), Age: 69.4 ± 8 Con: *n* = 36 (30.6% F) Age: 68.9 ± 7.6	**Duration** 6 weeks. **Intervention** Breathing exercises.	Usual care	Physical function (6MWT) Quality of life (SF-36) Anxiety and Depression (SDS, SAS)	Physical function improved with statistically differences in intervention group compared with baseline (*p* < 0.05), without statistically improvements in control group. Intervention group improved with statistically differences compared to control group (*p* < 0.05). Quality of life improved with statistically differences compared to baseline in intervention group (*p* < 0.05) and not on control group. Between group analysis found that intervention group improved with statistically differences in all items of SF-36 compared to control group (*p* < 0.05). Anxiety improved with statistically significant differences between groups favoring intervention (*p* < 0.05), but not depression.
Llurda-Almuzara et al. (2022) [47]	RCT	Adults with subacute COVID-19	*n* = 70 Int: *n* = 35, Age: 49.5 ± 13.7 Con: *n* = 35 Age: 55.1 ± 20.9	**Duration** 8 weeks. **Intervention** Aerobic, strength and breathing exercises. Telerehabilitation.	Usual care	Physical function (SPPB, 4MWT)	Physical function improved with moderate significant effects in intervention group compared to control.
Lobanov et al. (2022) [48]	RCT	Adults with subacute COVID-19	*n* = 23 Int: *n* = 14 Con: *n*= 9	**Duration** 2 weeks. **Intervention** Aerobic exercises in pool.	Exercise without pool.	Physical function (6MWT) Quality of life (EQ-5D) Dyspnea (BS)	Physical function improved with statistically significant differences compared to baseline (*p* = 0.047 both groups), with greater improvement in intervention group. Quality of life improved in anxiety/depression domain with statistically differences in control group (*p* = 0.043), but not in intervention group (*p* = 0.69). Dyspnea improved after treatment, but without statistical differences compared to baseline in any group.
Martín et al. (2021) [49]	Observational	Adults with subacute COVID-19	*n* = 27 Int: *n* = 14 (21.4% F), Age: 60.8 ± 10.4 Con: *n* = 13 (53.8% F), Age: 61.9 ± 10.7	**Duration** 6 weeks. **Intervention** Aerobic and strength exercises.	Usual Care	Physical function (1MSTST) Dyspnea (BS)	After treatment, statistically differences were found in 1min-STS favoring intervention group (*p* = 0.004). No differences were found in terms of dyspnea improvement (*p* = 0.560).
Nagy et al. (2022) [50]	RCT	Adults with subacute COVID-19	*n* = 52 Int: *n* = 26, Age: 40 ± 3.36 Con: *n* = 26, Age: 39.7 ± 3.55	**Duration** 6 weeks. **Intervention** Myofascial release and breathing exercises.	Breathing exercises	Dyspnea (mMRC) Physical function (6MWT) Fatigue (FSS)	Dyspnea, physical function, and fatigue improved with statistical differences compared to baseline in both groups (*p* < 0.05). Additionally, intervention group resulted in statistically significant differences compared to control (*p* < 0.001).
Nambi et al. (2022) [51]	RCT	Adults with subacute COVID-19	*n* = 76 Int: *n* = 38, Age: 63.2 ± 3.1 Con: *n* = 38 Age: 64.1 ± 3.2	**Duration** 8 weeks. **Intervention** Exercise at low intensity	Exercise at high intensity	Quality of life (SarQol)	Both groups improved quality of life after treatment with statistical differences compared to baseline (*p* = 0.001). However, patients allocated to low intensity group improved with better results in SarQol compared to baseline than those allocated to high intensity training.
Pehlivan et al. (2022) [52]	RCT	Adults with subacute COVID-19	*n* = 34 Int: *n* = 17 (18% F), Age: 50.76 (32–82) Con: *n* = 17 (35% F), Age: 43.24 (23–71)	**Duration** 6 weeks. **Intervention** Aerobic, strength and breathing exercises. Telerehabilitation.	Usual care	Physical function (TUG/SPPB) Dyspnea (mMRC) Fatigue (VAS) Quality of life (SGRQ) Depression (BDI)	Although both groups improved outcomes, intra-group differences were only found mMRC (*p* = 0.035), TUG (*p* = 0.005) and SGRQ (*p* = 0.002) at intervention group, while not statistically differences were found in control group at the end of treatment. Between-groups analysis revealed statistically significant differences in terms of SGRQ improvement favor to intervention (*p* = 0.042). No significant changes were found after treatment in depression levels neither intra-group or between group comparison.
Puchner et al. (2021) [53]	Observational	Adults with subacute COVID-19	*n* = 23 (30% F) Age: 57 ± 10	**Duration** 3–4 weeks. **Intervention** Aerobic, strength and breathing exercises. Nutritional and psychological counseling.	No control	Physical function (6MWT)	Physical function improved after treatment with statistically differences compared to baseline (*p* < 0.001).
Rodríguez-Blanco et al. (2021) [54]	RCT	Adults with subacute COVID-19	*n* = 36 Int: *n* = 18 (50% F), Age: 39.39 ± 11.74 Con: *n* = 18 (55.5% F), Age: 41.33 ± 12.13	**Duration** 1 week. **Intervention** Strength exercises. Telerehabilitation.	Usual care	Physical function (6MWT/30STS) Dyspnea (BS)	Intervention group improved physical function after treatment with statistically differences compared to usual care (*p* < 0.001). However, although dyspnea improved in intervention group and did not improve in control group after treatment, differences were not significant (*p* = 0.074).
Rutkowski et al. (2022) [55]	RCT	Adults with subacute COVID-19	*n* = 32 (68% F) Age: 57.8 ± 4.9	**Duration** 3 weeks. **Intervention** Virtual reality exercise	Exercise without virtual reality	Depression and Anxiety (HADS) Quality of life (WHOQOL-BREF) Physical function (6MWT)	Intervention group (*p* < 0.001) and control group (*p* < 0.05) improved anxiety and depression after treatment compared to baseline levels. No significant changes were found in any group in terms of quality-of-life improvement after treatment. Physical function improved in both groups. However, patients in intervention group showed more improvements in walked distance after treatment than control group.
Teixeira do Amaral et al. (2022) [56]	RCT	Adults with subacute COVID-19	*n* = 32 Int: *n* = 12, Age: 51.9 ± 10.2 Con: *n* = 20, Age: 53.3 ± 11.6	**Duration** 12 weeks. **Intervention** Aerobic and strength exercises. Telerehabilitation.	Usual care	Physical function (6MWT, TUG, 5TSTS)	Both groups all physical function outcomes compared to baseline, but without statistically significant differences within-group or between groups.
**Studies with long COVID-19 patients**							
Albu et al., 2022 [57]	Observational	Adults with long COVID-19	*n* = 40 (40% female) Mean Age: 52 ± 11.4 y/o	**Duration** 8 weeks **Intervention** Education Aerobic, strength and breathing exercises. Psychological counseling. **Intensity** Personalized according to patient status.	No control	Physical performance (SPPB) Fatigue (MFIS) Quality of life (WHOQOL-BREF)	After 8 weeks of rehabilitation, significant improvements in physical performance were found in SPPB compared to baseline with statistically differences (*p* = 0.001). Fatigue was improved after intervention with statistically differences for all measured domains (*p* = 0.001). Quality of life improved in physical, psychological, and environmental domains with statistical differences (*p* = 0.001), but not at social domain (*p* = 0.15).
Cahalan et al., 2022 [58]	Observational	Adults with long COVID-19	*n* = 27 (85% f) Mean age: 48.4 ± 10.1 y/o	**Duration** 10 weeks. **Intervention** Breathing exercises, psychological advice. Telerehabilitation. **Intensity** Not reported.	None	Dyspnea (C19YRS) Fatigue (C19YRS) Anxiety/Depression (C19YRS)	Statistical improvements were found after treatment in terms of dyspnea (*p* < 0.001), as well as in fatigue (*p* = 0.03). Although anxiety and depression improved after treatment, no significant differences were found (*p* = 0.08 for anxiety, *p* = 0.337 for depression).
Calvo-Paniagua 2022 [59]	Quasi-experimental	Adults with long COVID-19	*n* = 68 (61.8% f) Mean age: 48.5 ± 9.7 y/o	**Duration** 7 weeks. **Intervention** Aerobic, strength and breathing exercises. Telerehabilitation. **Intensity** Not reported.	None	Dyspnea (mMRC) Quality of life (SGRQ) Physical performance (6MWT)	Dyspnea improved significantly after intervention and at follow-up (*p* < 0.001). Quality of life improved significantly after intervention and at follow-up (*p* < 0.001). Physical performance improved with statistically differences after intervention and at follow-up (*p* < 0.001).
Compagno et al., 2022 [60]	Observational	Adults with long COVID-19	*n* = 30 (40% female) Mean Age: 58.37 ± 11.6 y/o	**Duration** 8–20 weeks **Intervention** Aerobic and strength exercises. Psychological counseling. **Intensity** Aerobic exercise at low and mid intensity. Strength at 30–50% 1RM.	No control	Quality of life (SF-36) Anxiety (SAS) Depression (SDS)	Quality of life improved after intervention with statistically differences (*p* < 0.05). Anxiety and depression improved with statistically differences after treatment (both *p* < 0.05).
Daynes et al., 2021 [61]	Observational	Adults with long COVID-19	*n* = 30 (48% female) Mean Age: 58 ± 16 y/o	**Duration** 6 weeks, with two supervised sessions per week. **Intervention** Aerobic and strength exercises. **Intensity** Not reported.	No control	Physical performance (ISWT) Fatigue (FACIT) Anxiety and depression (HADS) Quality of life (EQ-5D)	ISWT improved after treatment with statistically differences compared to baseline (*p* < 0.01). Fatigue improved with statistical differences at the end of treatment (*p* < 0.01), while anxiety and depression improved, but without statistically significant differences (*p* = 0.5 for anxiety and *p* = 0.1 for depression). Quality of life improved after treatment compared to baseline (*p* = 0.05).
Del Corral 2022 [62]	RCT	Adults with long COVID-19	G1: *n* = 22, mean age: 48.9 ± 8.3 y/o; 77% f G2: *n* = 22, mean age: 45.3 ± 12.8 y/o; 73% f G3: *n* = 22, mean age: 46.5 ± 9.6 y/o, 64% f G4: *n* = 22, mean age: 45 ± 10.2 y/o, 73% f	**Duration** 8 weeks. **Intervention** Group 1: Inspiratory breathing exercises. Group 2: Inspiratory and expiratory breathing exercises. Telerehabilitation. **Intensity** 20–80% of maximal inspiratory pressure	Group 3: Sham inspiratory exercises. Group 4: Sham inspiratory and expiratory exercises. Sham procedures were with device without resistance	Quality of life (EQ-5D) Physical performance (1MSTST) Anxiety/Depression (HADS)	All groups improved quality of life after intervention compared to baseline (*p* < 0.05), except group 4. At 4 weeks follow-up, no statistical differences were found between groups improving quality of life. Physical performance improved with large effects in intervention groups compared with sham groups after intervention (*p* < 0.01), but without differences when comparing both intervention groups. Differences were not found between groups after 4 weeks follow-up in terms of physical performance improving. Although all groups improved psychological status, no statistical differences were found across groups.
Estébanez-Pérez 2022 [63]	Quasi-Experimental	Adults with long COVID-19	*n* = 32 (71.9% f) Mean age: 45.93 ± 10.65 y/o	**Duration** 4 weeks. **Intervention** Aerobic and strength training. Telerehabilitation. **Intensity** Aerobic exercises at low to moderate intensity. Strength training not reported.	None	Physical performance (SPPB, 1MSTST)	1mSTS and SPPB improved with statistically significant effects after treatment (*p* < 0.05).
Groenveld 2022 [64]	Observational	Adults with long COVID-19	*n* = 47 (68% f) Mean age: 54 (21–70)	**Duration** 6 weeks **Intervention** Virtual reality-based exercise. Telerehabilitation. **Intensity** Adjusted to patient.	None	Fatigue (BS) Physical performance (6MWT, TUG, 30CST) Quality of life (SF-12, PHQ) Anxiety/Depression (HADS)	Fatigue improved with clinical differences after treatment (*p* = 0.03). Significant differences were found in 6MWT (*p* < 0.001) and 30CST (*p* = 0.02) after intervention. Three patients performed TUG instead of 6MWT, with improvements after treatment. Statistical differences were found improving quality of life for physical sphere (*p* < 0.049) and mental sphere (*p* < 0.01) measured with SF-12, as well as with PHQ (*p* = 0.04) Symptoms measured with HADS decreased, but without statistical differences (*p* = 0.08).
Hasenoehrl et al., 2022 [65]	Quasi-experimental	Adults with long COVID-19	Group 1 (mild symptoms): *n* = 10 (60% female), mean age: 42.9 ± 12.4 y/o Group 2 (severe symptoms): *n* = 18 (89% female), mean age: 47.4 ± 10.1 y/o	**Duration** 8 weeks of supervised strength training, 2 times per week **Intervention** Aerobic and strength exercises. **Intensity** Strength exercises performed at 7–10 RPE. Aerobic exercises at moderate intensity.	No control	Physical performance (6 MWT/30 STST)	Both groups improved significantly 30 STST (*p* < 0.001) and 6 MWT (*p* < 0.001) after intervention.
Jimeno-Almanzán et al., 2022 [36]	RCT	Adults with long COVID-19	*n* = 80 (69% female) Mean Age: 45.3 ± 8.0 y/o	**Duration** 8 weeks. **Intervention** G1: Strength and breathing exercises. G2: Strength exercises. G3: Breathing exercises. **Intensity** Strength at 50% 1 RM. Breathing exercises at 12–15 RPE.	G4: Usual care	Dyspnea (mMRC) Quality of life (SF-12) Anxiety and Depression (GAD-7/PHQ-9) Fatigue (FSS)	All outcomes improved in all study groups after intervention. After 8 weeks of intervention, no differences between groups were detected in mMRC, GAD-7 and SF-12. Fatigue and depression improved with differences in training groups (G1 and G2, *p* = 0.007). Breathing training group (G3) improved with differences in physical domain of SF-12 (*p* < 0.05). No relevant changes were observed in control group (G4) pre-post intervention.
Jimeno-Almanzán et al., 2022a [66]	RCT	Adults with long COVID-19	*n* = 39 (74.4% female) Mean Age: 45.2 ± 9.5 y/o	**Duration** 8 weeks. **Intervention** Strength exercises. **Intensity** 50% 1RM.	Usual care	Dyspnea (mMRC) Quality of life (SF-12) Anxiety and Depression (GAD-7/PHQ-9) Fatigue (FSS) Physical performance (5TSTST)	Intervention group resulted in statistically differences compared to control in physical domain of SF-12 (*p* = 0.024), fatigue (*p* < 0.05), depression symptoms (*p* = 0.021), and physical performance (*p* = 0.009). Although all studied outcomes improved in both groups, no statistical differences were found in other outcomes such as dyspnea improvement or anxiety.
Lloyd-Evans 2022 [67]	Observational	Adults with long COVID-19	*n* = 110 (68.1% f) Mean age: 46.3 ± 10.8	**Duration** 8–12 weeks **Intervention** Aerobic and strength exercises. Telerehabilitation. **Intensity** Not reported.	None	Quality of life (EQ-5D)	Statistically significant differences were found improving quality of life (*p* < 0.01).
McNarry 2022 [68]	RCT	Adults with long COVID-19	*n* = 148 (111 int, 86% f/37 con, 95% f) Mean age: 46.76 ± 12.03 (int)/46.13 ± 12.73 (con)	**Duration** 8 weeks, unsupervised. **Intervention** Breathing exercises. Telerehabilitation. **Intensity** 80% of sustained maximal inspiratory pressure.	Usual care	Quality of life (K-BILD) Dyspnea (TDI)	Although quality of life improved within-group, no statistically significant differences were found between groups. Dyspnea improved with statistical differences favoring intervention compared to control (*p* = 0.005).
Nopp et al., 2022 [69]	Observational	Adults with long COVID-19	*n* = 58 (43.1% female) Mean Age: 46.8 ± 12.6 y/o	**Duration** 6 weeks. **Intervention** Aerobic, strength and breathing exercises. **Intensity** Not reported.	No Control	Physical performance (6 MWT/1 MSTST) Dyspnea (mMRC) Quality of life (EQ-5D) Fatigue (FAS)	After intervention, patients improved 6 MWT and 1 MSTST with statistical differences (*p* < 0.001). Dyspnea improved with statistical differences compared to baseline (*p* < 0.001). Quality of life improved after treatment (*p* < 0.001). Fatigue improved after treatment with statistical differences (*p* < 0.001).
Okan 2022 [70]	RCT	Adults with long COVID-19	*n* = 52 (26 int, 42.3% f/26 con, 53.8% f) Mean age: 48.85 ± 10.85 (int)/52.19 ± 14.84 (con)	**Duration** 5 weeks, one session supervised. **Intervention** Aerobic and breathing exercises. Telerehabilitation. **Intensity** Aerobic exercises at moderate intensity. Breathing not reported.	Usual care	Dyspnea (mMRC) Physical performance (6 MWT) Quality of life (SGRQ)	Both groups improved dyspnea. However, it was significantly lower in intervention group than in control group (*p* < 0.001). Quality of life improved with statistical differences in intervention group compared to control after treatment (*p* < 0.001). Physical performance improved with statistically significant differences in intervention group compared to control (*p* < 0.001).
Philip 2022 [71]	RCT	Adults with long COVID-19	*n* = 150 (81% f) Mean age: 49 ± 12	**Duration** 6 weeks. **Intervention** Breathing exercises. Telerehabilitation. **Intensity** Not reported.	Usual care	Quality of life (SF-36) Dyspnea (DS-12) Anxiety (GAD-7)	Intervention group improved mental component of SF-36 with statistical differences compared to control (*p* = 0.047), while no differences in physical component (*p* = 0.54). Dyspnea improved in both groups compared to baseline, but without differences between groups (*p* = 0.38). Although anxiety improved in both groups, no statistical differences were found between group (*p* = 0.085).

**Abbreviations:** F (Female); DS-12 (Dyspnea Severity Index 12); EQ-5D (EuroQol 5D); 6MWT (6 Minute Walking Test); RCT (Randomized Controlled Trial); MBS (Modified Borg Scale); 30STS (30 s Sit-to-Stand Test); MD12 (Multidimensional Dyspnea 12); BS (Borg Scale); NRS (Numeric Rating Scale); mMRC (Modified Medical Research Council Scale); BFI (Brief Fatigue Inventory); PHQ-9 (Patient Health Questionnaire-9); GAD-7 (General Anxiety Disorders 7); TUG (Time up and go Test); SF-12 (Short Form 36); SDS (Self-Rating Depression Scale); SAS (Self-Rating Anxiety Scale); SPPB (Short Physical Performance Battery); 4MWT (4 min walking test); 1MSTST (1 min Sit to Stand Test); FSS (Fatigue Severity Scale); SarQol (Sarcopenia and Quality of life Questionnaire); VAS (Visual Analogue Scale); SGRQ (Saint George Respiratory Questionnaire); BDI (Beck Depression Inventory); HADS (Hospital Anxiety and Depression Scale); WHOQOL-BREF (World Health Organization Quality of Life Questionnaire); 5TSTS (5 Times Sit-to-stand); MFIS (Modified Fatigue Impact Scale); C19YRS (Covid 19 Yorkshire Rehabilitation Scale); SF-36 (Short Form 36); ISWT (Incremental Shuttle Walking Test); FACIT (Functional Assessment of Chronic Illness Therapy); FSS (Fatigue Severity Scale); K-BILD (King’s Brief Interstitial Lung Disease Questionnaire); TDI (Transition Dyspnea Index); FAS (Fatigue Assessment Scale).

## Data Availability

The data presented in this study are available on request from the corresponding authors.

## Supplementary Materials

The following supporting information can be downloaded at: https://www.mdpi.com/article/10.3390/biomedicines11082213/s1, Figure S1: Subgroup analysis of Randomized Controlled Trials; Figure S2: Subgroup analysis of Observational studies; Figure S3: Outlier studies analysis for RCT; Figure S4: Outlier studies analysis for observational studies; Figure S5: Leave-one-out analysis in RCT studies; Figure S6: Leave-one-out analysis in observational studies; Figure S7: Baujat plot in RCT studies; Figure S8: Baujat plot in observational studies; Figure S9: Funnel plot in RCT studies; Figure S10: Funnel plot in observational studies; Table S1: PRISMA 2020 Checklist; Table S2: Begg and Egger test for publication bias in included studies. Reference [19] is cited in Supplementary Materials.

## Appendix A

### Pubmed Search String

(Rehab* OR exercise OR training OR physical therapy OR breathing OR pulmonary rehabilitation OR rehabilitation OR pulmonary OR respiratory OR education) AND (post-coronavirus OR post-covid OR long covid OR post-covid-19 OR persistent covid OR long-covid-19 OR covid-19 symptoms OR post covid-19 OR post coronavirus OR post covid OR long-covid OR post-acute COVID-19 syndrome OR COVID-19 post-intensive care syndrome OR COVID-19 OR SARS-CoV-2 OR coronavirus OR post-acute COVID-19) AND (Quality of life [MeSH] OR health-related quality of life OR Functional status [MeSH] OR function OR functionally OR disability evaluation [MeSH] OR outcome assessment [MeSH] OR patient-reported outcome measure [MeSH] OR disability).
