# Supplementary material for: Efficacy of Pulmonary Rehabilitation in Post-COVID-19: A Systematic Review and Meta-Analysis
Source: Biomedicines. 2023 Aug 7;11(8):2213. doi: 10.3390/biomedicines11082213 (PMC10452542; doi:10.3390/biomedicines11082213)
Supplement: Supplementary file 1 [file biomedicines-11-02213-s001.zip › Suplementary Material Figures and Table S2.pdf]

Figure S1: Subgroup analysis of Randomized Controlled Trials [36,39,40,41,42,46,47,50,52,54,56,66,68,70,71]

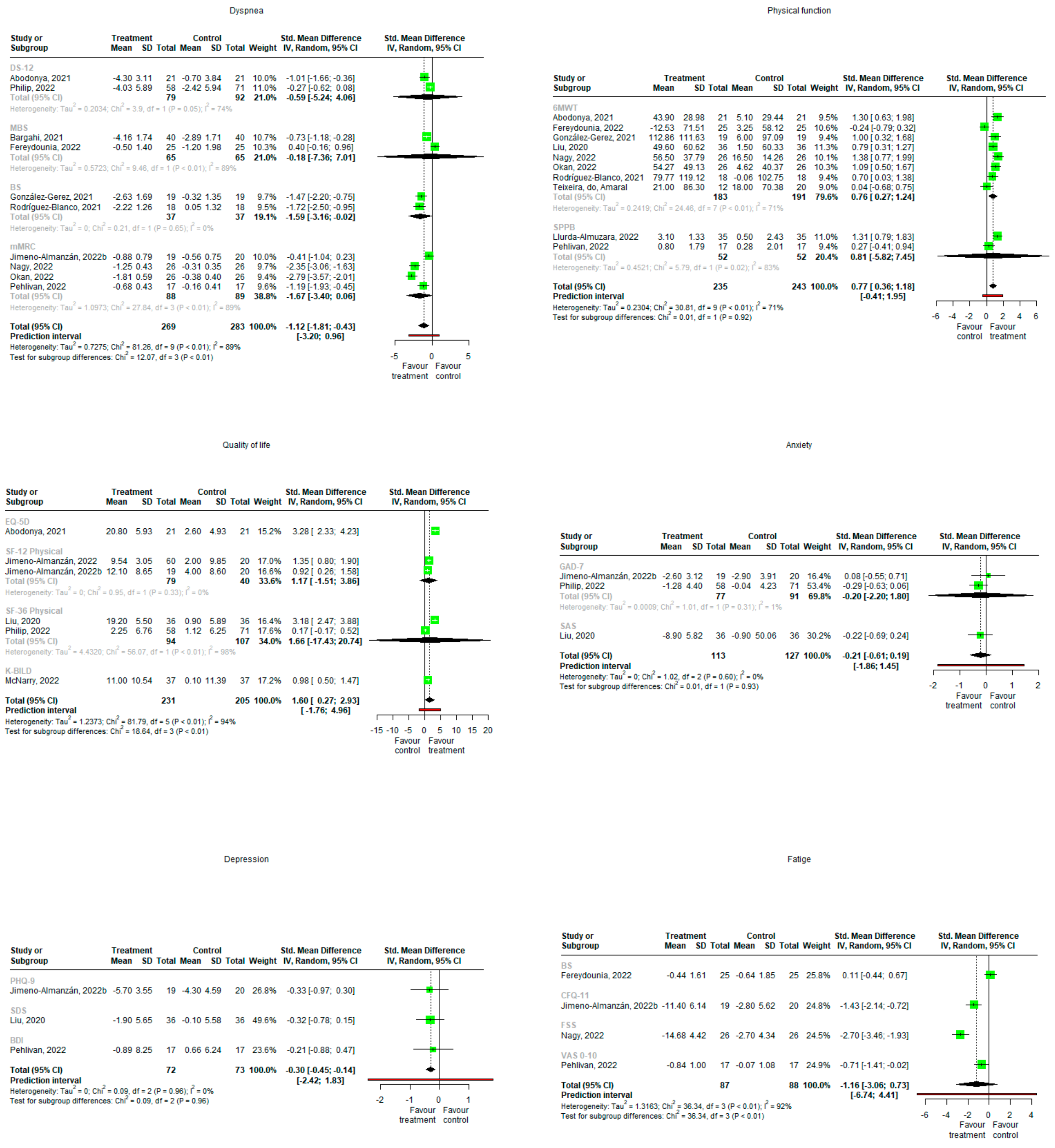

Figure S2: Subgroup analysis of Observational studies [43,44,53,57,59,60,61,63,64,67,69].

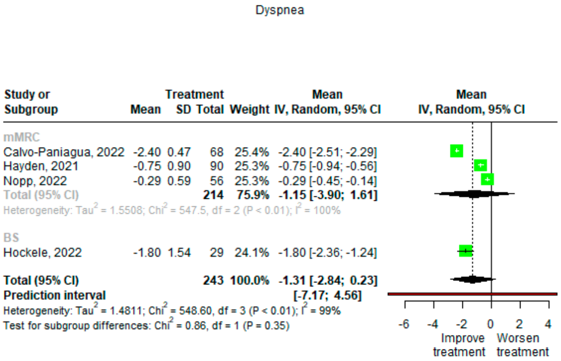

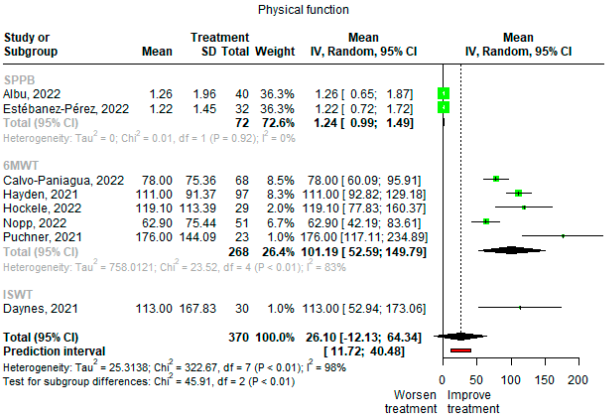

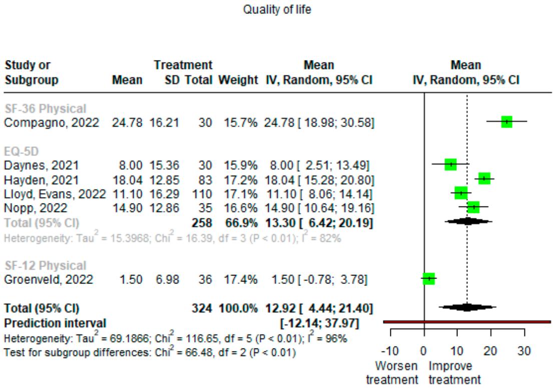

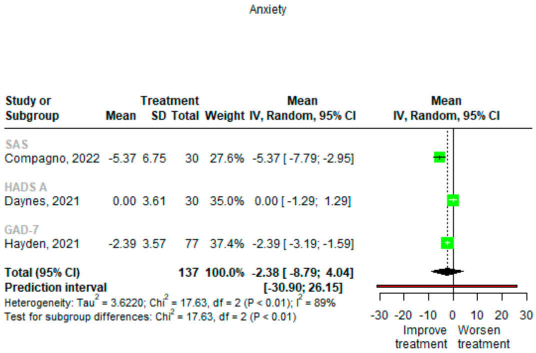

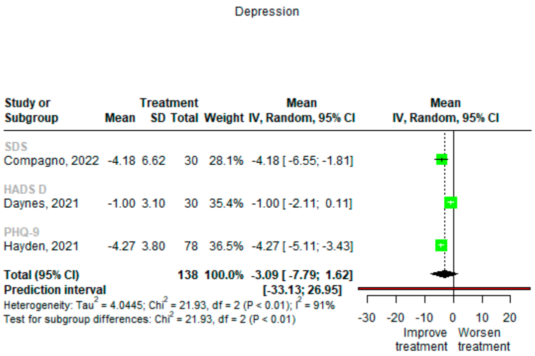

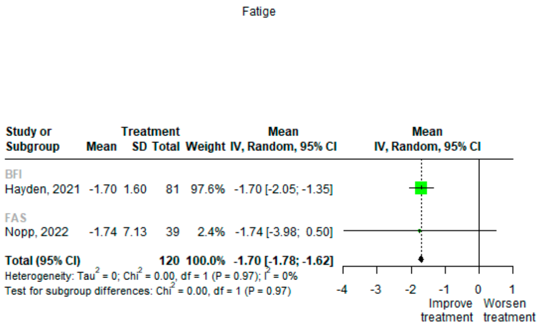

Figure S3: Outlier studies analysis for RCT [36,39,40,41,42,46,47,50,52,54,56,66,68,70,71].

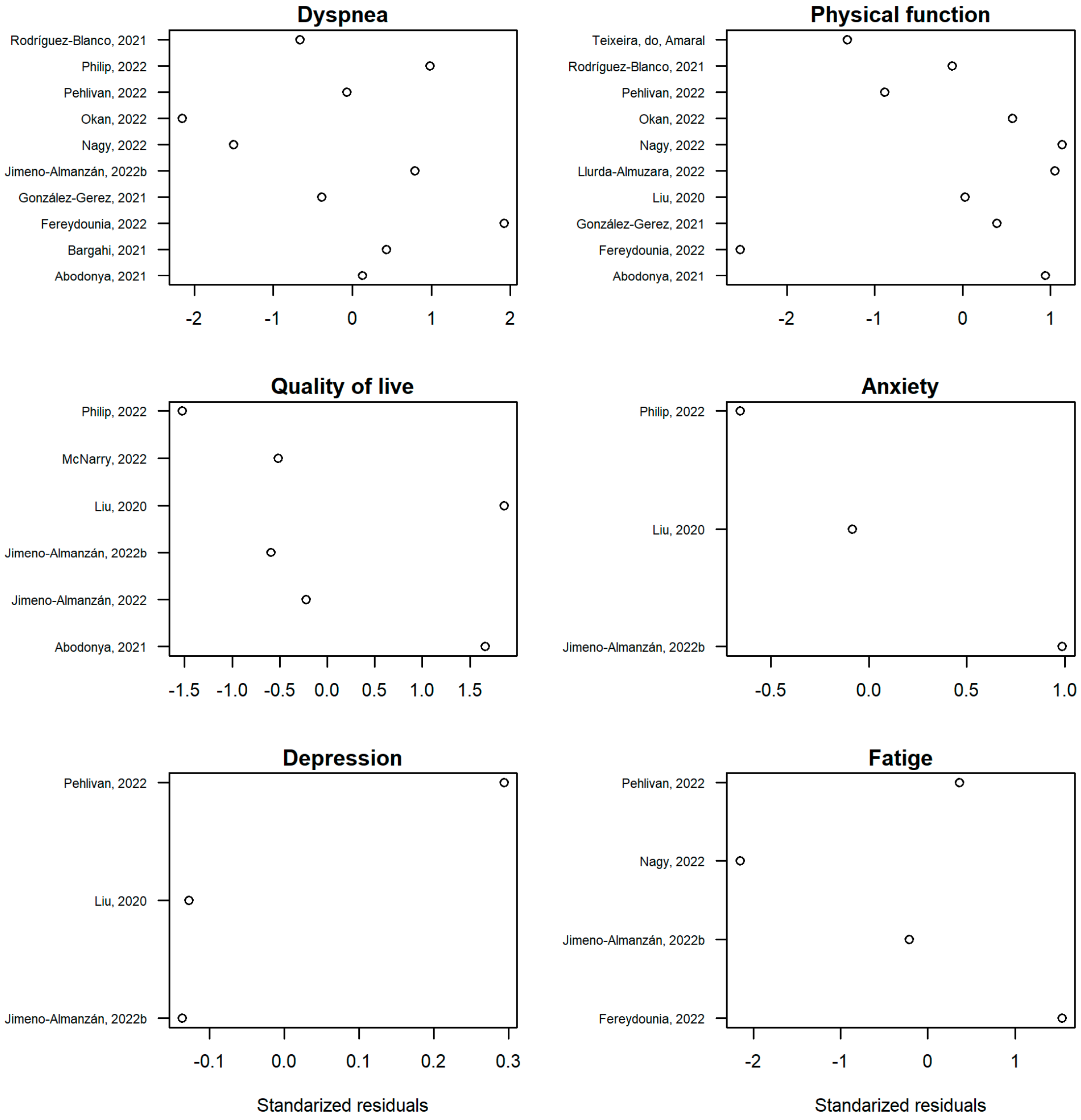

Figure S4: Outlier studies analysis for observational studies [43,44,53,57,59,60,61,63,64,67,69].

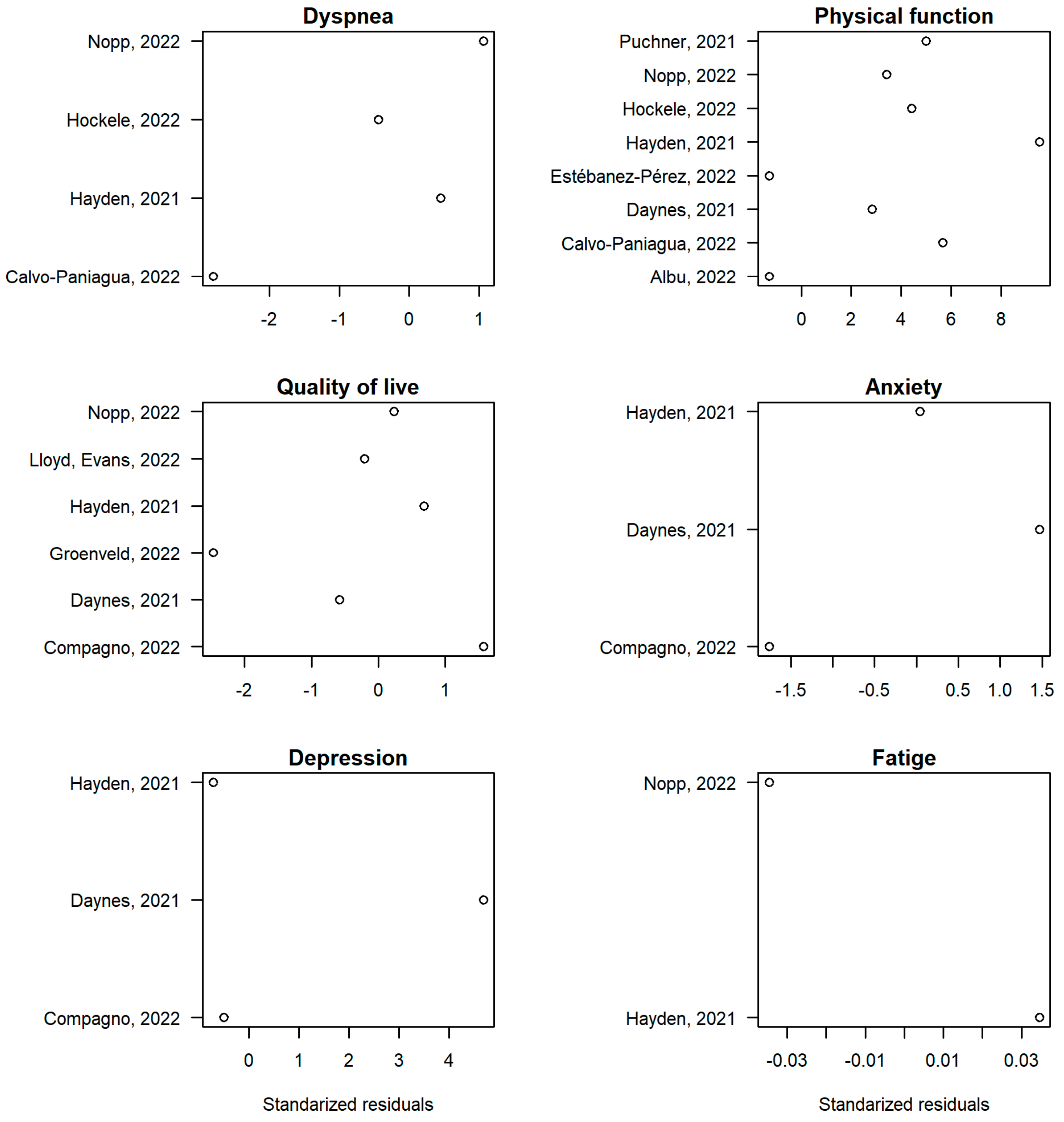

Figure S5: Leave-one-out analysis in RCT studies [36,39,40,41,42,46,47,50,52,54,56,66,68,70,71]

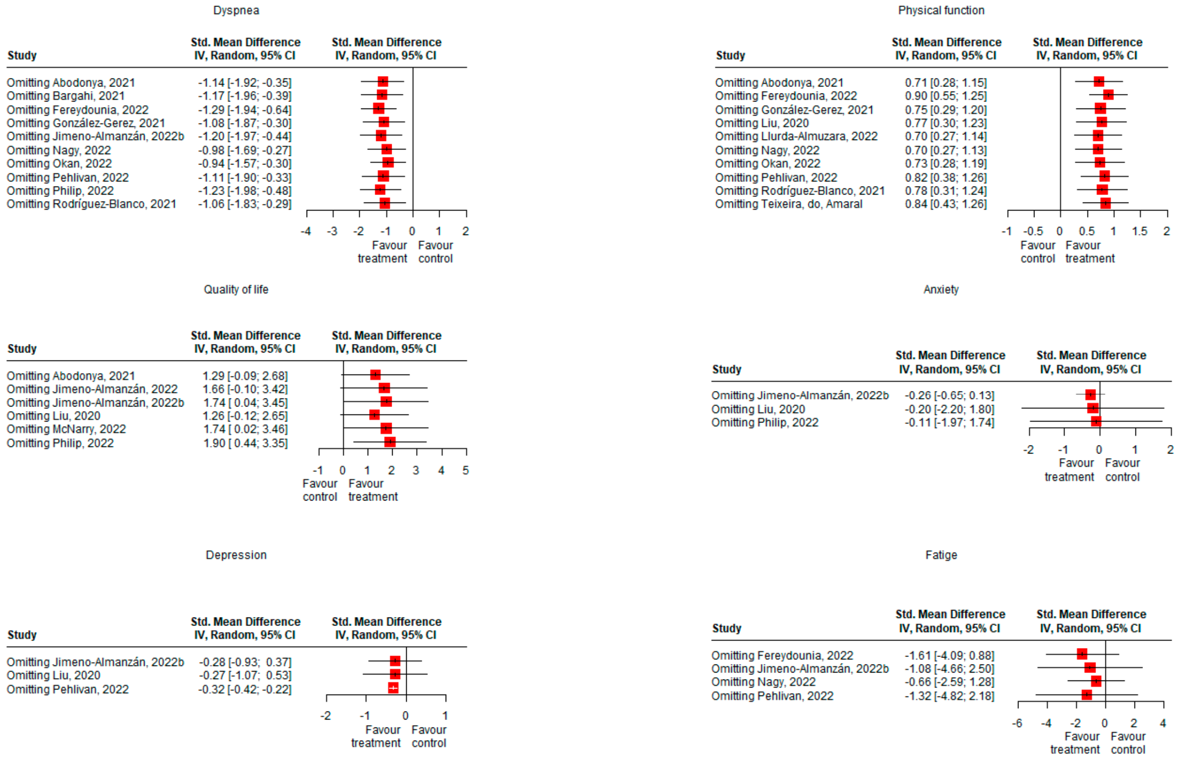

Figure S6: Leave-one-out analysis in observational studies [43,44,53,57,59,60,61,63,64,67,69].

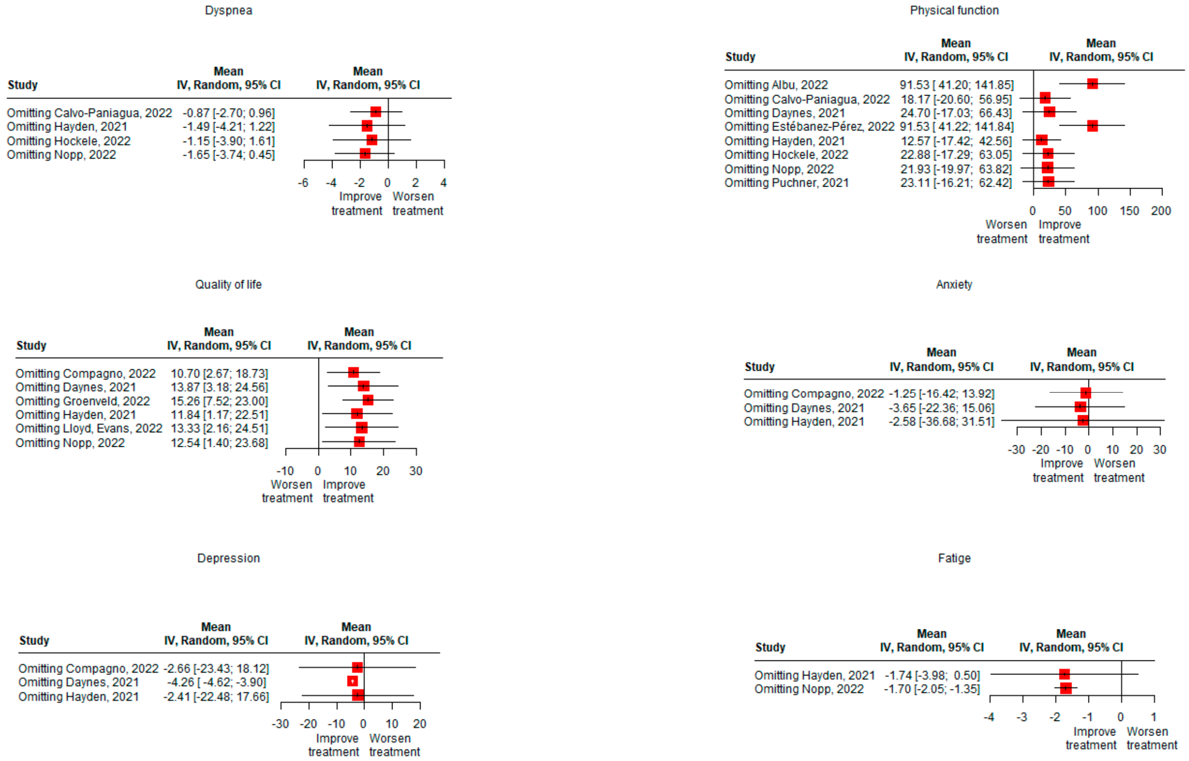

Figure S7: Baujat plot in RCT studies [36,39,40,41,42,46,47,50,52,54,56,66,68,70,71]

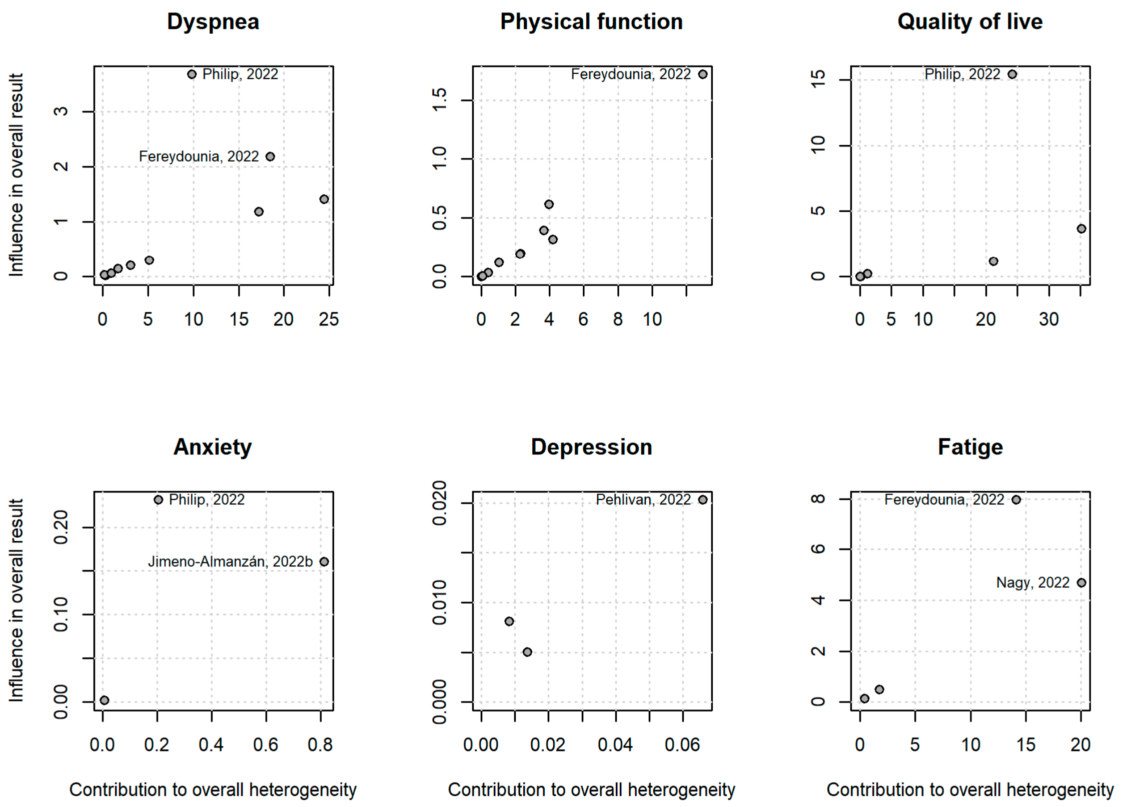

Figure S8: Baujat plot in observational studies [43,44,53,57,59,60,61,63,64,67,69].

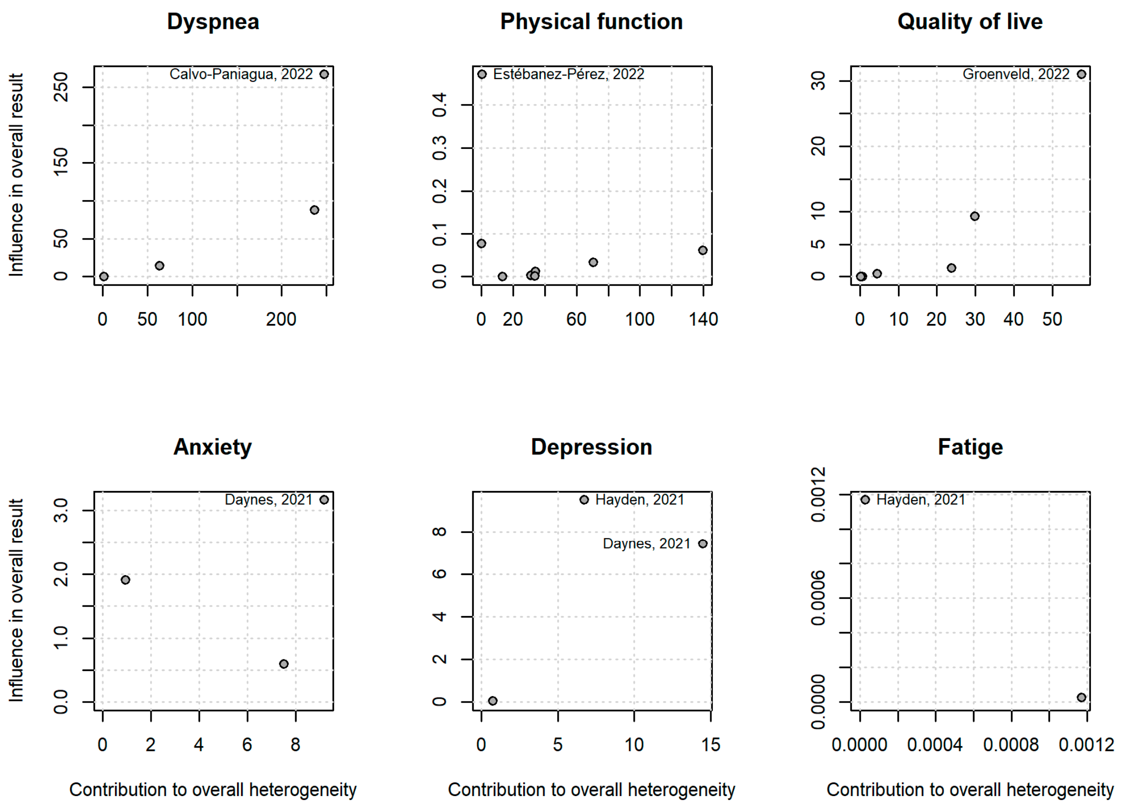

Figure S9: Funnel plot in RCT studies [36,39,40,41,42,46,47,50,52,54,56,66,68,70,71]

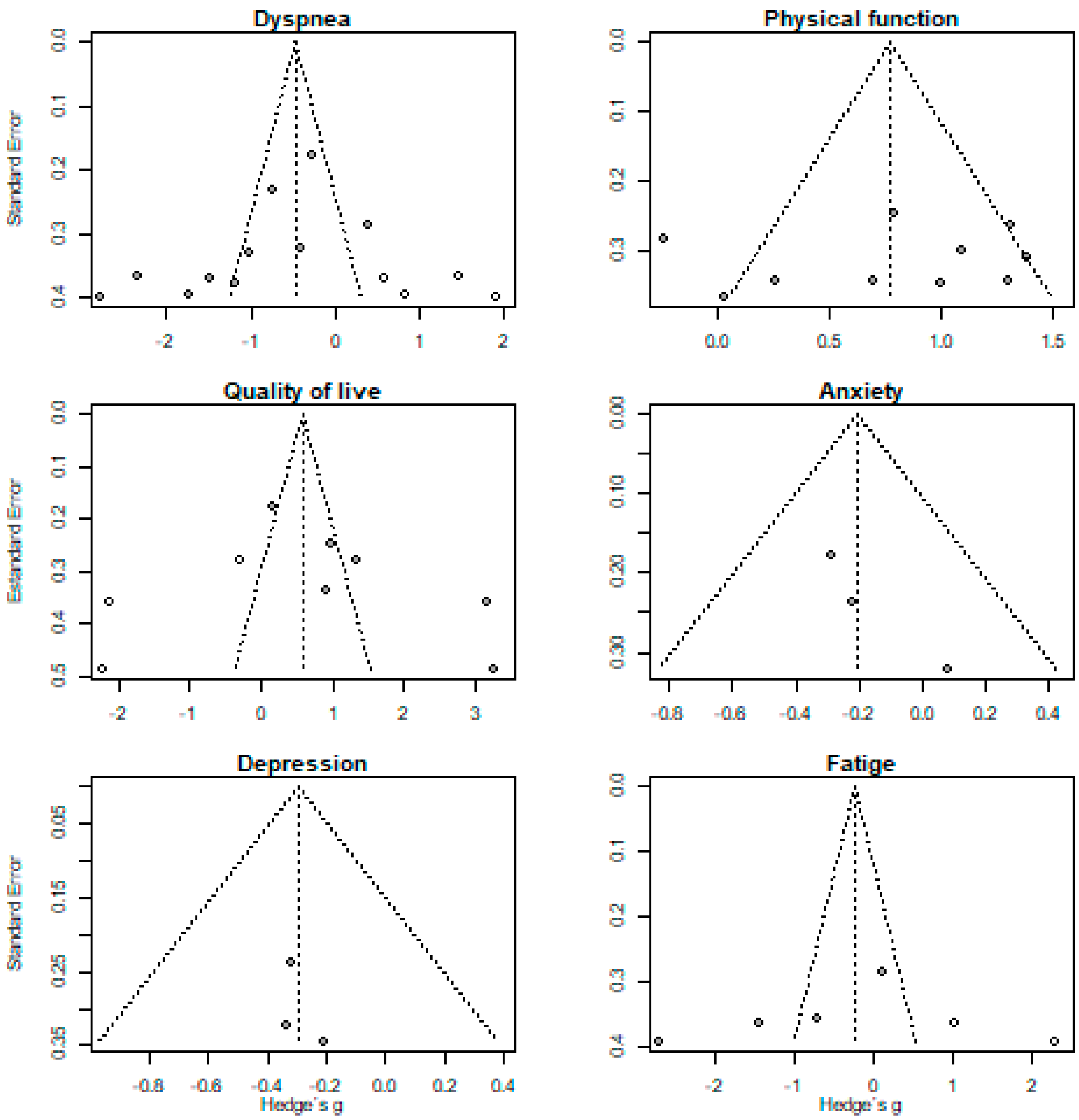

Figure S10: Funnel plot in observational studies [43,44,53,57,59,60,61,63,64,67,69].

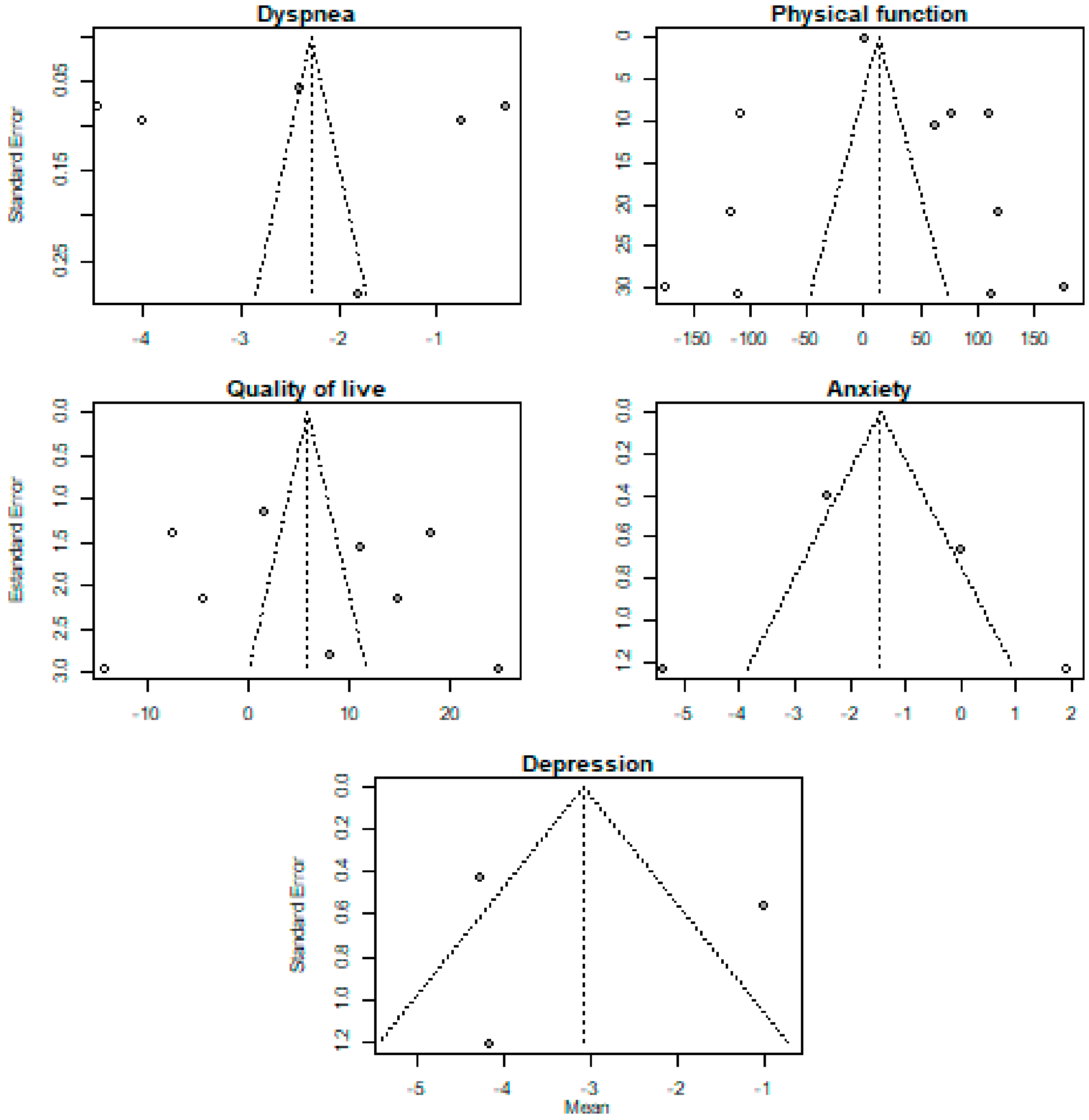

Table S2: Begg and Egger test for publication bias in included studies [36,39-44,46,47,50,52-54,56,57,59-61,63,64,66-71]

**RCTs**	**Begg test**	**Egger test**
Dyspnea	Kendall's τ=-0.689, p=0.005	t(8)=-2.802, p=0.023
Physical function	Kendall's τ=-0.289, p=0.291	t(8)=-0.473, p=0.649
Quality of live	Kendall's τ=0.6, p=0.136	t(4)=3.405, p=0.027
Anxiety	Kendall's τ=1, p=0.333	t(1)=3.2, p=0.193
Depression	Kendall's τ=0.333, p=>0.999	t(1)=0.688, p=0.616
Fatige	Kendall's τ=-1, p=0.083	t(2)=-3.144, p=0.088
**Observational studies**		
Dyspnea	Kendall's τ=0, p=>0.999	t(2)=-0.316, p=0.782
Physical function	Kendall's τ=0.071, p=0.905	t(6)=6.107, p=0.001
Quality of live	Kendall's τ=0.2, p=0.719	t(4)=1.14, p=0.318
Anxiety	Kendall's τ=-0.333, p=>0.999	t(1)=-0.967, p=0.511
Depression	Kendall's τ=0.333, p=>0.999	t(1)=-0.305, p=0.811
Fatige	Kendall's τ=-1, p=>0.999	

t(df): t statistic (degrees of freedom).
Significant if p<0.05 (shown in red).
